# Association between dry eye symptoms and suicidal ideation in a Korean adult population

**DOI:** 10.1371/journal.pone.0199131

**Published:** 2018-06-20

**Authors:** Sun-Bi Um, Hyungseon Yeom, Na Hyun Kim, Hyeon Chang Kim, Hyung Keun Lee, Il Suh

**Affiliations:** 1 Department of Public Health, Yonsei University Graduate School, Seoul, Republic of Korea; 2 Department of Preventive Medicine, Yonsei University College of Medicine, Seoul, Republic of Korea; 3 Institute of Vision Research, Department of Ophthalmology, Yonsei University College of Medicine, Seoul, Republic of Korea; Xiamen University, CHINA

## Abstract

**Purpose:**

This study assessed the association of dry eyes with depression and suicidal ideation in a Korean adult population.

**Methods:**

Data from 16408 participants (6972 men and 9436 women) aged ≥ 19 years included in the fifth Korea National Health and Nutrition Examination Survey, conducted from 2010–2012, were analyzed. For dry eyes, surveys of previous diagnosis of dry eye disease (DED) by an ophthalmologist and experience of subjective dry eye symptoms were separately used. Diagnosis of depression and suicidal ideation were obtained via responses to an interviewer-assisted questionnaire, and questions were asked in a closed-ended response format. Logistic regression was used to examine the associations between dry eyes, depression, and suicidal ideation.

**Results:**

DED diagnosis exhibited an odds ratio (OR) of 1.32 (95% confidence interval [CI] 1.11–1.57) for depression and 1.24 (95% CI 1.05–1.48) for suicidal ideation compared to those without DED, after adjusting for sex, age, education, occupation, household income, body mass index, smoking behavior, alcohol consumption, physical activity, hypertension, diabetes, dyslipidemia, thyroid diseases, major cardiovascular disease, and cancer. Similarly, the adjusted OR (95% CI) of dry eye symptoms was 1.50 (95% CI 1.30–1.73) for depression and 1.47 (95% CI 1.27–1.70) for suicidal ideation.

**Conclusion:**

Our findings suggest that dry eyes (either DED diagnosis or dry eye symptoms) may be associated with the prevalence of depression and suicidal ideation in the Korean adult population.

## Introduction

Worldwide, more than one million people die by suicide every year [1], and suicide rates are expected to increase from 1.8% in 1998 to 2.4% in 2020 [2]. The suicide rate of South Korea is highest among the Organization for Economic Cooperation and Development (OECD) countries recently. As the burden due to suicide increased, suicide became a major health issue in South Korea [3]. While suicide may be associated with biological, behavioral, physical, socio-cultural, and environmental factors, mental illness (including depression, bipolar disease, schizophrenia, and others) is the main cause of suicide [4–6]. Nevertheless, while the link between suicide and mental illness is well established, studies have yet to thoroughly investigate whether ocular diseases are linked with suicide.

Dry eye disease (DED) is one of the most frequently encountered ocular morbidities worldwide. The prevalence of DED is estimated to range from 4.3% to 73.5% for either clinically diagnosed DED or cases that experienced symptoms and is comparably higher in Asian populations than in Western populations [7–13]. Vision plays an important role in almost every task that humans perform, at all stages of life, regardless of age. The health of the human eye depends on the flow of tears, which provide constant moisture and lubrication to maintain vision and comfort. However, once tear flow is impaired, the human eye can experience symptoms of redness, stinging, burning, or a scratchy sensation, which lead to eye fatigue, visual disturbance, and even impaired quality of life (QOL) [13–18]. Additionally, patients with DED frequently report significant disturbances in their psychiatric state, showing symptoms of anxiety and depression [19–21].

Many suicidal attempts happen impulsively in moments of crisis, along with a breakdown in one’s ability to deal with life stresses, such as chronic pain and illness [22]. However, beyond depression, relatively few studies investigated whether prolonged symptoms of DED may cause suicidal ideation. Therefore, we aimed to examine the association between DED, depression, and suicidal ideation using nationally representative data targeting the entire Korean population. We hypothesized that equivalent results would be observed as reported by previous studies concerning the association between DED and depression, and reports of suicidal ideation would be higher in participants with DED.

## Materials and methods

### Study population

This study used data from the fifth Korea National Health and Nutrition Examination Survey (KNHANES-V), which was conducted from 2010–2012. The KNHANES is a nationally representative cross-sectional survey of the non-institutionalized civilian population. The survey is repeated annually by the Korea Centers for Disease Control and Prevention (KCDC). A complex, stratified, multistage probability sampling design based on region and households was applied in this survey to represent the national population of South Korea. The survey consisted of three parts: a health interview survey, a health examination survey, and a nutrition survey. Survey questionnaires were self-administered or conducted by trained staff members depending on the section, and examinations were performed by highly trained medical personnel. Further details of the survey have been described elsewhere [23]. The survey procedures were carried out in accordance with the Declaration of Helsinki, and participants who were willing to participate in the survey were required to sign informed consent forms approved by the Institutional Review Board of the KCDC. Data are available from the KCDC (https://knhanes.cdc.go.kr/knhanes/eng/index.do). The design of this study was approved by the Institutional review board of Yonsei University Graduate School of Public Health in Seoul, Korea.

A total of 18571 participants aged 19 years or older were initially included over the three years for analyses. We further excluded 1921 participants that were lacking information in dry eyes, depression diagnosis, suicidal ideation, and other covariates. Since previous studies indicated that patients taking antidepressants had a higher chance of developing DED [10, 24, 25], we additionally excluded 242 participants who were receiving treatment for depression. Lastly, after excluding non-responses on dry eye questionnaires, 6972 men and 9436 women were analyzed in relation to their DED diagnosis, while 6718 men and 9146 women were analyzed according to their dry eye symptoms. All analyses were performed separately according to the presence of DED diagnosis and dry eye symptoms (not mutually exclusive).

### Assessment of dry eyes

Examination for eye diseases was conducted by ophthalmologists designated by the Korean Ophthalmological Society (KOS). In order to ensure accurate examinations, ophthalmologists were educated and trained twice a year by the KCDC and the KOS. In 2010, the KNHANES introduced dry eye questionnaires to evaluate the prevalence of dry eyes. Due to the survey design, presence of DED and dry eye symptoms were observed by closed-ended response questionnaires. Ophthalmologists interviewed each participant on whether they had been diagnosed with DED by an ophthalmologist before. To increase accuracy of the data collected, participants were also asked whether they experienced frequent symptoms of dryness or irritation of the eye. Participants who responded “yes” to the above questionnaires were either assigned to DED diagnosis or dry eye symptoms. Those who answered “no” were classified as a control.

### Assessment of depression and suicidal ideation

Diagnosis of depression was assessed by interviewing each participant on whether they had previously been diagnosed with depression by a psychiatrist. To increase the accuracy of the data collected, participants were first asked whether they had been diagnosed with depression so far which was followed up “by a psychiatrist.” Suicidal ideation was assessed by asking participants whether they had thought about dying within the last year.

### Covariates

Age, sex, socioeconomic status including education, occupation, and household income, body mass index (BMI), smoking behavior, alcohol consumption, physical activity, diagnosis of hypertension, diabetes, dyslipidemia, thyroid diseases, major CVD, and cancer were considered as covariates in the present analyses. Age and BMI were analyzed as continuous variables. Education was classified according to the highest graduate level of school (middle school/high school/college), occupation was classified according to the standard occupation classification, and household income was classified in quartiles. Smoking behavior was categorized as non-smoker (never smoked for their entire life) versus former or current smoker. Alcohol consumption was categorized as non-drinker (never consumed alcohol for their entire life and/or within the past year) versus drinker (consumption of alcohol once per week or less or twice per week or more). Physical activity was categorized according to frequency of exercise: none (no physical activity within the past week) versus at least once per week or more. Diagnosis of other diseases were obtained by interviewer-assisted questionnaire.

### Statistical Analysis

All statistical analyses in this study were conducted using sampling weights assigned to each participant provided by the KNHANES. Student’s t-test and chi-square test were used to analyze the general characteristics of the study population according to presence of dry eyes (DED diagnosis and dry eye symptoms). Serial multiple logistic regression models were used to examine the independent associations between dry eye, depression, and suicidal ideation. All analyses were separated by sex. A *P*-value < 0.05 was considered statistically significant. All statistical analyses were performed using SAS version 9.2 (SAS Institute, Inc. Cary, NC).

## Results

### Characteristics of the study population according to the presence of dry eyes

DED diagnosed men had a higher household income level, higher frequency of white-collar and lower frequency in pink, green, and grey-collar occupations. They showed a lower frequency of alcohol consumption and higher frequency of physical activity compared to men without DED diagnosis (Table 1). DED diagnosed men had a higher frequency of dyslipidemia, and for mental health components, perceived stress, depression diagnosis, and suicidal ideation did not differ between the two groups. The comparison of men with dry eye symptoms and without, showed a similar distribution with DED diagnosis. Interestingly, men with dry eye symptoms noted a significantly higher frequency in the mental health components. Men experiencing dry eye symptoms reported significantly higher levels of stress perception, (28.7% versus 24.0%, p = 0.03), prevalence of depression (9.3% versus 6.1%, p = 0.003) and suicidal ideation (12.0% versus 9.0%, p = 0.04) than men without symptoms.

**Table 1:** Characteristics of men according to the presence of dry eye.

Variables	DED diagnosis			Dry eye symptoms		
	No (n = 6598)	Yes (n = 374)	p value	No (n = 5946)	Yes (n = 772)	p value
Age, years	44.3 ± 16.4	46.1 ± 16.1	0.09	44.3 ± 15.4	45.5 ± 16.2	0.12
Body mass index, kg/m^2^	24.1 ± 3.2	24.2 ± 2.9	0.68	24.1 ± 3.4	23.8 ± 3.0	0.05
House income						
Lowest	1177 (17.8)	59 (15.8)	0.02	1481 (20.8)	410 (20.1)	0.74
Low	1719 (26.1)	95 (25.4)		1822 (25.6)	516 (25.4)	
High	1860 (28.2)	97 (25.9)		1900 (26.7)	544 (26.7)	
Highest	1842 (27.9)	123 (32.9)		1908 (26.8)	565 (27.8)	
Educational level						
≤ Middle school	1970 (29.8)	89 (23.8)	0.08	1782 (29.9)	220 (28.5)	0.14
High school	2353 (35.7)	139 (37.2)		2132 (35.9)	264 (34.2)	
≥ College	2275 (34.5)	146 (39.0)		2032 (34.2)	288 (37.3)	
Occupation						
Manager	1072 (16.2)	84 (22.5)	<0.001	981 (16.5)	148 (19.2)	0.01
Clerk	679 (10.3)	38 (10.2)		600 (10.1)	79 (10.2)	
Service and sales	743 (11.3)	31 (8.3)		671 (11.3)	68 (8.8)	
Agricultural, forestry and fishery	744 (11.3)	19 (5.1)		673 (11.3)	60 (7.8)	
Craft, equipment, machine operating and assembling	1227 (18.6)	47 (12.6)		1100 (18.5)	129 (16.7)	
Elementary	507 (7.7)	29 (7.8)		468 (7.9)	61 (7.9)	
Housewife, students, etc.	1626 (24.6)	126 (33.7)		1453 (24.4)	227 (29.4)	
Alcohol consumption						
None	1072 (16.2)	79 (21.1)	0.02	975 (16.4)	138 (17.9)	0.45
≤ 1 time/week	2992 (45.3)	185 (49.5)		2699 (45.4)	362 (46.9)	
≥ 2 times/week	2534 (38.4)	110 (29.4)		2272 (38.2)	272 (35.2)	
Smoking behavior						
None	1215 (18.4)	78 (20.9)	0.07	1107 (18.6)	139 (18)	0.36
Former	2669 (40.5)	185 (49.5)		2404 (40.4)	355 (46)	
Current	2714 (41.1)	111 (29.7)		2435 (41.0)	278 (36)	
Physical activity						
No	3949 (59.9)	203 (54.3)	0.02	3542 (59.6)	459 (59.5)	0.32
≥ 1 days/week	2649 (40.2)	171 (45.7)		2404 (40.4)	313 (40.5)	
Hypertension						
No	5098 (77.3)	275 (73.5)	0.16	4607 (77.5)	569 (73.7)	0.21
Yes	1500 (22.7)	99 (26.5)		1339 (22.5)	203 (26.3)	
Dyslipidemia						
No	5984 (90.7)	314 (84.0)	0.001	5395 (90.7)	677 (87.7)	0.01
Yes	614 (9.3)	60 (16.0)		551 (9.3)	95 (12.3)	
Diabetes						
No	5944 (90.1)	334 (89.3)	0.64	5349 (90.0)	697 (90.3)	0.76
Yes	654 (9.9)	40 (10.7)		597 (10.0)	75 (9.7)	
Thyroid disease						
No	6521 (98.8)	369 (98.7)	0.40	5881 (98.9)	757 (98.1)	0.13
Yes	77 (1.2)	5 (1.3)		65 (1.1)	15 (1.9)	
Major CVD ^a^						
No	6357 (96.4)	355 (94.9)	0.26	5727 (96.3)	738 (95.6)	0.56
Yes	241 (3.7)	19 (5.1)		219 (3.7)	34 (4.4)	
Cancer						
No	6398 (97.0)	358 (95.7)	0.76	5773 (97.1)	738 (95.6)	0.35
Yes	200 (3.0)	16 (4.3)		173 (2.9)	34 (4.4)	
Perceived stress						
No and mild	5085 (77.1)	286 (76.5)	0.45	4612 (77.6)	567 (73.5)	0.03
Moderate to severe	1513 (22.9)	88 (23.5)		1334 (22.4)	205 (26.6)	
Depression diagnosis						
No	6154 (93.3)	340 (90.9)	0.19	5561 (93.5)	695 (90.0)	0.004
Yes	444 (6.7)	34 (9.1)		385 (6.5)	77 (10.0)	
Suicidal ideation						
No	5950 (90.2)	336 (89.8)	0.56	5381 (90.5)	673 (87.2)	0.03
Yes	648 (9.8)	38 (10.2)		565 (9.5)	99 (12.8)	

DED: dry eye disease, CVD: cardiovascular diseases.

Data are expressed as means ± standard deviation or numbers (%).

^a^ Major CVD includes myocardial infarction, and stroke.

DED diagnosed women were more frequent in agricultural workers, and showed significant differences in smoking behavior, perceived stress, and depression diagnosis compared to women without DED diagnosis, although no significant difference was observed in suicidal ideation (Table 2). Regarding dry eye symptoms, significant differences were observed in mental health components, similarly to men. Women with either DED diagnosis or dry eye symptoms had a higher frequency of dyslipidemia and thyroid disesases. Women with dry eye symptoms were likely to perceive more stress (35.7% versus 28.1%, p<0.001), have depression (23.2% versus 17.5%, p<0.001), and suicidal ideation (22.0% versus 16.6%, p<0.001) than women without symptoms.

**Table 2:** Characteristics of women according to the presence of dry eye.

Variables	DED diagnosis			Dry eye symptoms		
	No (n = 8119)	Yes (n = 1317)	p value	No (n = 7111)	Yes (n = 2035)	p value
Age, years	46.2 ± 16.7	46.1 ± 16.1	0.79	46.3 ± 16.5	46.1 ± 16.5	0.74
Body mass index, kg/m^2^	23.3 ± 3.6	23.0 ± 3.5	0.04	23.3 ± 3.7	23.1 ± 3.7	0.08
House income						
Lowest	1706 (21.0)	253 (19.2)	0.78	1481 (20.8)	410 (20.1)	0.74
Low	2070 (25.5)	336 (25.5)		1822 (25.6)	516 (25.4)	
High	2155 (26.5)	359 (27.3)		1900 (26.7)	544 (26.7)	
Highest	2188 (26.9)	369 (28.0)		1908 (26.8)	565 (27.8)	
Educational level						
≤ Middle school	3426 (42.2)	521 (39.6)	0.32	2980 (41.9)	857 (42.1)	0.52
High school	2543 (31.3)	437 (33.2)		2245 (31.6)	640 (31.4)	
≥ College	2150 (26.5)	359 (27.3)		1886 (26.5)	538 (26.4)	
Occupation						
Manager	794 (9.8)	126 (9.6)	0.01	692 (9.7)	194 (9.5)	0.07
Clerk	511 (6.3)	96 (7.3)		436 (6.1)	152 (7.5)	
Service and sales	1067 (13.1)	162 (12.3)		934 (13.1)	251 (12.3)	
Agricultural, forestry and fishery	576 (7.1)	44 (3.3)		490 (6.9)	107 (5.3)	
Craft, equipment, machine operating and assembling	203 (2.5)	38 (2.9)		181 (2.5)	53 (2.6)	
Elementary	768 (9.5)	107 (8.1)		673 (9.5)	174 (8.6)	
Housewife, students, etc.	4200 (51.7)	744 (56.5)		3705 (52.1)	1104 (54.3)	
Alcohol consumption						
None	3020 (37.2)	502 (38.1)	0.67	2665 (37.5)	771 (37.9)	0.22
≤ 1 times/week	4388 (54)	724 (55)		3824 (53.8)	1107 (54.4)	
≥ 2 times/week	711 (8.8)	91 (6.9)		622 (8.7)	157 (7.7)	
Smoking behavior						
None	7219 (88.9)	1207 (91.6)	0.01	6320 (88.9)	1845 (90.7)	0.22
Former	437 (5.4)	65 (4.9)		383 (5.4)	100 (4.9)	
Current	463 (5.7)	45 (3.4)		408 (5.7)	90 (4.4)	
Physical activity						
No	5556 (68.4)	914 (69.4)	0.94	4887 (68.7)	1384 (68.0)	0.26
≥ 1 days/week	2563 (31.6)	403 (30.6)		2224 (31.3)	651 (32.0)	
Hypertension						
No	6277 (77.3)	991 (75.2)	0.57	5519 (77.6)	1529 (75.1)	0.29
Yes	1842 (22.7)	326 (24.8)		1592 (22.4)	506 (24.9)	
Dyslipidemia						
No	7276 (89.6)	1081 (82.1)	<0.001	6368 (89.6)	1730 (85.0)	<0.001
Yes	843 (10.4)	236 (17.9)		743 (10.4)	305 (15.0)	
Diabetes						
No	7542 (92.9)	1207 (91.6)	0.81	6613 (93.0)	1867 (91.7)	0.22
Yes	577 (7.1)	110 (8.4)		498 (7.0)	168 (8.3)	
Thyroid disease						
No	7683 (94.6)	1191 (90.4)	<0.001	6735 (94.7)	1872 (92.0)	<0.001
Yes	436 (5.4)	126 (9.6)		376 (5.3)	163 (8.0)	
Major CVD ^a^						
No	7955 (98.0)	1292 (98.1)	0.88	6971 (98.0)	1996 (98.1)	0.95
Yes	164 (2.0)	25 (1.9)		140 (2.0)	39 (1.9)	
Cancer						
No	7797 (96.0)	1260 (95.7)	0.57	6822 (95.9)	1955 (96.1)	0.96
Yes	322 (4.0)	57 (4.3)		289 (4.1)	80 (3.9)	
Menopausal status						
Premenopausal	3823 (47.1)	566 (43.0)	0.25	3345 (47)	889 (43.7)	0.17
Postmenopausal	4296 (52.9)	751 (57.0)		3766 (53)	1146 (56.3)	
Perceived stress						
No and mild	5882 (72.4)	898 (68.2)	0.001	5201 (73.1)	1363 (67)	< .001
Moderate to severe	2237 (27.6)	419 (31.8)		1910 (26.9)	672 (33)	
Depression diagnosis						
No	6582 (81.1)	1018 (77.3)	0.01	5804 (81.6)	1565 (76.9)	< .001
Yes	1537 (18.9)	299 (22.7)		1307 (18.4)	470 (23.1)	
Suicidal ideation						
No	6741 (83.0)	1070 (81.2)	0.09	5956 (83.8)	1605 (78.9)	< .001
Yes	1378 (17.0)	247 (18.8)		1155 (16.2)	430 (21.1)	

DED: dry eye disease, CVD: cardiovascular diseases.

Data are expressed as mean (standard deviation) or numbers (%).

^a^ Major CVD includes myocardial infarction, and stroke.

### Association between dry eyes and depression

Table 3 shows the odds ratio (OR) and 95% confidence interval (CI) of depression when unadjusted and adjusted for age, socioeconomic status (education, occupation, household income), BMI, smoking behavior, alcohol consumption, physical activity, history of hypertension, diabetes, dyslipidemia, thyroid diseases, major CVD, and cancer. Regardless of sex, participants diagnosed with DED were associated with a higher prevalence of depression (OR 1.32; 95% CI 1.11–1.57), compared to non-DED participants. After stratifying for sex, the association remained significant for women (OR 1.31; 95% CI 1.08–1.57), though was no longer significant in men (OR 1.32; 95% CI 0.84–2.09).

**Table 3:** Association between dry eye and depression in the overall participants and by sex.

Dry eye	Overall				Men				Women			
	Total	Depression	Odds ratio (95% CI)		Total	Depression	Odds ratio (95% CI)		Total	Depression	Odds ratio (95% CI)	
			Unadjusted	Adjusted ^a^			Unadjusted	Adjusted ^b^			Unadjusted	Adjusted ^c^
Dry eye disease diagnosis												
No	14717	1981	1.00 [Reference]	1.00 [Reference]	6598	444	1.00 [Reference]	1.00 [Reference]	8119	1537	1.00 [Reference]	1.00 [Reference]
Yes	1691	333	1.68 (1.43–1.98)	1.32 (1.11–1.57)	374	34	1.35 (0.86–2.10)	1.32 (0.84–2.09)	1317	299	1.28 (1.07–1.54)	1.31 (1.08–1.57)
Dry eye symptoms												
No	13057	1692	1.00 [Reference]	1.00 [Reference]	5946	385	1.00 [Reference]	1.00 [Reference]	7111	1307	1.00 [Reference]	1.00 [Reference]
Yes	2807	547	1.68 (1.42–1.99)	1.50 (1.30–1.73)	772	77	1.58 (1.16–2.16)	1.55 (1.13–2.13)	2035	470	1.44 (1.23–1.68)	1.47 (1.25–1.72)

Odds ratio and confidence intervals were estimated by considering sampling weights.

^a^ Adjusted for age, sex, education, occupation, household income, body mass index, smoking behavior, alcohol consumption, physical activity, hypertension, diabetes, dyslipidemia, thyroid diseases, major cardiovascular diseases (myocardial infarction and stroke), and cancer.

^b^ Adjusted for age, education, occupation, household income, body mass index, smoking behavior, alcohol consumption, physical activity, hypertension, diabetes, dyslipidemia, thyroid diseases, major cardiovascular diseases, and cancer.

^c^ Adjusted for age, education, occupation, household income, body mass index, smoking behavior, alcohol consumption, physical activity, menopausal status, hypertension, diabetes, dyslipidemia, thyroid diseases, major cardiovascular diseases, and cancer.

Similar patterns were observed for participants experiencing dry eye symptoms. Overall, participants with dry eye symptoms showed an OR of 1.50 (95% CI 1.30–1.73) for depression than those without dry eye symptoms. The ORs for depression with dry eye symptoms were 1.55 (95% CI 1.13–2.13) for men and 1.47 (95% CI 1.25–1.72) for women, respectively.

### Association between dry eyes and suicidal ideation

Table 4 shows the OR and 95% CI of suicidal ideation when unadjusted and adjusted for potential confounders. Overall, participants diagnosed with DED had an OR of 1.24 (95% CI 1.05–1.48) for suicidal ideation than non-DED participants. Women (OR 1.26; 95% CI 1.05–1.52) with DED were also more likely to have suicidal ideation than those without DED, however in men (OR 1.17; 95% CI 0.76–1.80) a significant association did not exist.

**Table 4:** Association between dry eye and suicidal ideation in the overall participants and by sex.

Dry eye	Overall				Men				Women			
	Total	Suicidal ideation	Odds ratio (95% CI)		Total	Suicidal ideation	Odds ratio (95% CI)		Total	Suicidal ideation	Odds ratio (95% CI)	
			Unadjusted	Adjusted ^a^			Unadjusted	Adjusted ^b^			Unadjusted	Adjusted ^c^
Dry eye disease diagnosis												
No	14717	12691	1.00 [Reference]	1.00 [Reference]	6598	5950	1.00 [Reference]	1.00 [Reference]	8119	6741	1.00 [Reference]	1.00 [Reference]
Yes	1691	1406	1.39 (1.17–1.65)	1.24 (1.05–1.48)	374	336	1.14 (0.74–1.74)	1.17 (0.76–1.80)	1317	1070	1.17 (0.98–1.40)	1.26 (1.05–1.52)
Dry eye symptoms												
No	13057	11337	1.00 [Reference]	1.00 [Reference]	5946	5381	1.00 [Reference]	1.00 [Reference]	7111	5956	1.00 [Reference]	1.00 [Reference]
Yes	2807	2278	1.61 (1.39–1.86)	1.47 (1.27–1.70)	772	673	1.38 (1.03–1.85)	1.40 (1.05–1.87)	2035	1605	1.43 (1.21–1.68)	1.49 (1.27–1.76)

Odds ratio and confidence intervals were estimated by considering sampling weights.

^a^ Adjusted for age, sex, education, occupation, household income, body mass index, smoking behavior, alcohol consumption, physical activity, hypertension, diabetes, dyslipidemia, thyroid diseases, major cardiovascular diseases (myocardial infarction and stroke), and cancer.

^b^ Adjusted for age, education, occupation, household income, body mass index, smoking behavior, alcohol consumption, physical activity, hypertension, diabetes, dyslipidemia, thyroid diseases, major cardiovascular diseases, and cancer.

^c^ Adjusted for age, education, occupation, household income, body mass index, smoking behavior, alcohol consumption, physical activity, menopausal status, hypertension, diabetes, dyslipidemia, thyroid diseases, major cardiovascular diseases, and cancer.

Participants with dry eye symptoms had an OR of 1.47 (95% CI 1.27–1.70) for suicidal ideation compared to the control group. Compared to those without dry eye symptoms, those with dry eye symptoms had a higher OR (95% CI) for suicidal ideation (1.40 (1.05–1.87) in men, and 1.49 (1.27–7.76) in women, respectively).

## Discussion

In the Korean population, we found that a previous diagnosis of DED, or frequent experiences of dry eye symptoms were significantly associated with depression and suicidal ideation. However, for the association between DED diagnosis and suicidal ideation, statistical significance was observed in the overall participants and for women, when separated by sex in the analysis. According to previous studies [7, 26, 27], women often reported a higher prevalence of DED than men did, and our results support these findings. Suicide rates in South Korea have remained the highest among the OECD countries for 10 consecutive years since 2002, and were reported to be more than double the average of all OECD countries in 2012 (28.1 versus 12.1 for 100,000 people) [1]. Therefore, the high suicide rate in South Korea and a high prevalence of DED, which has been noted in Asian populations, makes South Korea a suitable country in which to analyze the association between dry eye and suicidal ideation.

The results on the relationship between DED and depression in this study are consistent with those in previous studies, although, relatively few studies examined the relationship between DED and suicidal ideation. One case-control study reported a higher prevalence of anxiety and depression in DED patients than the control group [19]. Additionally, the Beijing Eye study determined that depression was more prevalent in older patients with DED than those without [28]. Epidemiological studies have revealed that major depressive disorders are strong predictors of suicide attempts compared to other psychiatric disorders, including anxiety, agitation, and poor behavioral control; and 60% of those who commit suicide have a depressive disorder [29, 30]. Depression can also affect an individual to think about suicide. For example, impaired health behavior and low physical health status can cause suicidal ideation through depressive symptoms [5]. In light of the findings in our study, we suggest that depression may act as a mediator between DED and suicidal ideation.

To assess the role of depressive symptoms in the association between dry eye and suicidal ideation, we conducted further analyses. Additional adjustment for depressive symptoms weakened the association between DED diagnosis and suicidal ideation, but did not affect the association between dry eye symptoms and suicide ideation (S1 and S2 Tables). These findings suggest that depressive symptoms may not be the only mechanism which links dry eye and suicidal ideation. Further studies are required to elucidate the role of depression between dry eye and suicidal ideation.

Prolonged dry eye symptoms can lead to chronic pain, which in turn may cause feelings of depression and suicidal thoughts. Patients with chronic pain often develop complications of physical dysfunction and an altered psychological state, which negatively affect a person’s everyday living and QOL [31, 32]. In a parallel manner, persistent dry eye symptoms may induce a depressive mood. A previous study demonstrated that the presence of ocular surface symptoms decreased the ability to perform daily activities, work capacities, and emotional well-being [33]. Other studies have shown that people who experience chronic pain are more likely to develop depression than those who do not and that suicidal ideation is highly co-morbid [22, 34, 35]. Additionally, patients with chronic illness and pain have an increased risk of suicidal ideation and suicide attempt, even after adjusting for mental disorders [22]. Accordingly, the chronic impact of dry eye symptoms on various aspects of life may lead to the development of depression, and as the symptoms worsen, suicide might arise as an alternative to escape unbearable pain. Unfortunately, we could not evaluate these mediations, because the KNHANES did not assessed chronic pain in this period.

Our results showed that experiencing dry eye symptoms was more strongly associated with suicidal ideation than DED diagnosis. Among those who experienced dry eye symptoms, 1,375 participants were diagnosed with DED, while 1,440 participants were not. Several participants diagnosed with DED would have been receiving ophthalmic treatments to alleviate their pain, but participants who were undiagnosed and who had dry eye symptoms may have developed depression and suicidal thoughts. Furthermore, the total number of participants with dry eye symptoms almost doubled the number of participants with DED diagnosis. Thus, the greater sample size in this study might have increased the statistical significance of the association between dry eye symptoms and suicidal ideation. Therefore, according to the results and by the nature of the data analyzed, dry eye symptoms may have revealed a higher association with suicidal ideation compared to DED diagnosis.

Previous studies have demonstrated that DED patients have significantly lower QOL and vision-related QOL. In both the Women's Health Study and Physician's Health Study, DED patients demonstrated problems with reading, carrying out professional work, using a computer, watching TV, and driving during the day and night [16]. Another study that assessed general QOL in DED patients using the Short Form 36 Health Survey, which is a survey for measuring an individual’s overall wellbeing not specific to dry eyes, indicated that DED negatively affects everyday life by disrupting daily activities, and even causes great deterioration in mental health [15]. This study also demonstrated that in mild DED, a patient has the potential ability to overcome the dry eye symptoms, but this becomes limited as the severity of DED symptoms increases. In a utility assessment study using a time-trade-off method, participants responded that living 10 years with severe DED is comparable to living 1.6 years without DED [18]. Other studies have indicated that the presence of DED greatly reduces QOL and that a reduced QOL is associated with suicidal ideation and attempt [36, 37].

Proven risk factors of DED may be relevant to suicide. The Osaka study found that low physical activity and sedentary behavior were associated with DED [38]. Low physical activity was also linked with suicidal ideation. One study noted that low physical activity was correlated with suicidal ideation, and an absence of regular walking increased suicidal ideation [39]. Interestingly, diabetes [40], long working hours[41], smoking[42], air pollution[43], etc. have all been identified as risk factors of both DED and suicide.

Sleep disorders, such as insomnia or nightmare, are associated with the risk of suicide [44–46]. Sleep disorders are frequently observed in patients with DED, suggesting a possibility of sleep disorder as a confounder between DED and suicidal ideation. [47, 48]. In the KNHANES-V data, there was only information of sleep duration. However, our results did not change after additionally adjusting sleep duration (S3 and S4 Tables).

Although our results showed that participants with DED were more likely to experience depression and suicidal ideation, previous studies have indicated that patients with depression are susceptible to DED. Consistent results on the relationship between DED and the use of antidepressants have been reported in the literature [10, 25]. A cross-sectional study of psychiatric patients noted a higher prevalence of DED among patients with depression and/or anxiety disorders [49]. This study further observed that DED was associated with duration of psychiatric disease and use of antidepressant medications. Another study found that approximately 37% of participants who experienced symptoms of DED were taking antidepressants [7]. In our study, however, we excluded participants receiving depression treatments in order to assess the direct relationships between DED, depression, and suicidal ideation.

Our study has several limitations that need to be considered when interpreting the results. First, the association between DED and depression or suicidal ideation cannot be identified as causal due to the cross-sectional study design. Second, diagnosis of DED and dry eye symptoms were assessed by interviews rather than objective measurements. However, a survey conducted by the Korean Corneal Disease Study Group stated that Korean corneal subspecialists use either the diagnostic criteria of the International Dry Eye Workshop or the Dysfunctional Tear Syndrome Study Group guidelines to diagnose DED [38]. This statement provides evidence that participants diagnosed with DED were at least examined under internationally accepted criteria. Recent studies established the significance of using short questionnaires for determining the presence of DED in large epidemiological studies where objective examinations may not be practical [50], and demonstrated that asking about dryness and irritation was equivalent to asking a patient about up to 16 symptoms of DED [27]. Moreover, the questionnaires in the KNHANES were equivalent to one of the most frequently used short series of DED [39]. There are few evidences of the effects of the duration and severity of DED on depression or suicidal ideations. Unfortunately, KNHANES data do not have data of the duration or severity of DED, so we could not provide more informative evidences. Finally, the diagnosis of depression and suicidal ideation were obtained by interview using a single questionnaire. The validity and reliability of psychometric measures cannot be guaranteed. However, to increase the accuracy, participants were asked whether they have been diagnosed with depression “by a psychiatrist”. Despite these limitations, this is one of the first studies to report an association between dry eye and suicidal ideation using a large nationwide dataset with sufficient statistical power. The results of our analyses suggest that dry eye symptoms can be an aggravating factor for depression and suicidal ideation.

In conclusion, dry eye symptoms were associated with higher risks of depression and suicidal ideation in the Korean adult population. Our results suggest that DED should be viewed not only as an eye disorder, but also as a condition that affects mental health. Ophthalmologists may provide better treatment to patients with DED by evaluating their psychiatric status. However, further study is required to verify the prospective causal effect of DED on depression and suicidal ideation in a large population-based sample.

## Supporting information

S1 Table The results of multivariable logistic regression analyses for depression and suicidal ideation with DED diagnosis. (DOCX)

S2 Table The results of multivariable logistic regression analyses for depression and suicidal ideation with dry eye symptoms. (DOCX)

S3 Table The association between dry eye and suicidal ideation after adjusting depressive symptoms. (DOCX)

S4 Table The association between dry eye and suicidal ideation after adjusting sleep duration. (DOCX)

## References

[1] World Health Organization. Preventing suicide: A global imperative 2014. Available from: http://www.who.int/mental_health/suicide-prevention/world_report_2014/en/.

[2] Patton GC, Coffey C, Sawyer SM, Viner RM, Haller DM, Bose K, et al Global patterns of mortality in young people: a systematic analysis of population health data. Lancet. 2009;374(9693):881–92. Epub 2009/09/15. doi: 10.1016/S0140-6736(09)60741-8 . 19748397 10.1016/S0140-6736(09)60741-8

[3] Kim S-W, Yoon J-S. Suicide, an Urgent Health Issue in Korea. Journal of Korean Medical Science. 2013;28(3):345–7. doi: 10.3346/jkms.2013.28.3.345 PubMed PMID: PMC3594595. 23487589 10.3346/jkms.2013.28.3.345 PMC3594595

[4] Cheatle MD. Assessing suicide risk in patients with chronic pain and depression. The Journal of family practice. 2014;63(6 Suppl):S6–s11. Epub 2014/07/26. . 25061631

[5] Ro J, Park J, Lee J, Jung H. Factors that affect suicidal attempt risk among korean elderly adults: a path analysis. Journal of preventive medicine and public health = Yebang Uihakhoe chi. 2015;48(1):28–37. Epub 2015/02/06. doi: 10.3961/jpmph.14.030 . 25652708 10.3961/jpmph.14.030 PMC4322516

[6] Subramaniam M, Abdin E, Seow EL, Picco L, Vaingankar JA, Chong SA. Suicidal ideation, suicidal plan and suicidal attempts among those with major depressive disorder. Annals of the Academy of Medicine, Singapore. 2014;43(8):412–21. Epub 2014/09/24. . 25244990

[7] Chia EM, Mitchell P, Rochtchina E, Lee AJ, Maroun R, Wang JJ. Prevalence and associations of dry eye syndrome in an older population: the Blue Mountains Eye Study. Clinical & experimental ophthalmology. 2003;31(3):229–32. Epub 2003/06/06. . 12786773 10.1046/j.1442-9071.2003.00634.x

[8] Lee AJ, Lee J, Saw SM, Gazzard G, Koh D, Widjaja D, et al Prevalence and risk factors associated with dry eye symptoms: a population based study in Indonesia. The British journal of ophthalmology. 2002;86(12):1347–51. Epub 2002/11/26. ; PubMed Central PMCID: PMCPmc1771386. 12446361 10.1136/bjo.86.12.1347 PMC1771386

[9] Lin PY, Tsai SY, Cheng CY, Liu JH, Chou P, Hsu WM. Prevalence of dry eye among an elderly Chinese population in Taiwan: the Shihpai Eye Study. Ophthalmology. 2003;110(6):1096–101. Epub 2003/06/12. doi: 10.1016/S0161-6420(03)00262-8 . 12799232 10.1016/S0161-6420(03)00262-8

[10] Schaumberg DA, Dana R, Buring JE, Sullivan DA. Prevalence of dry eye disease among US men: estimates from the Physicians' Health Studies. Archives of ophthalmology. 2009;127(6):763–8. Epub 2009/06/10. doi: 10.1001/archophthalmol.2009.103 ; PubMed Central PMCID: PMCPmc2836718. 19506195 10.1001/archophthalmol.2009.103 PMC2836718

[11] Um SB, Kim NH, Lee HK, Song JS, Kim HC. Spatial epidemiology of dry eye disease: findings from South Korea. International journal of health geographics. 2014;13:31 Epub 2014/08/17. doi: 10.1186/1476-072X-13-31 ; PubMed Central PMCID: PMCPmc4139141. 25128034 10.1186/1476-072X-13-31 PMC4139141

[12] Viso E, Rodriguez-Ares MT, Gude F. Prevalence of and associated factors for dry eye in a Spanish adult population (the Salnes Eye Study). Ophthalmic epidemiology. 2009;16(1):15–21. Epub 2009/02/05. doi: 10.1080/09286580802228509 . 19191177 10.1080/09286580802228509

[13] Lekhanont K, Rojanaporn D, Chuck RS, Vongthongsri A. Prevalence of dry eye in Bangkok, Thailand. Cornea. 2006;25(10):1162–7. Epub 2006/12/19. doi: 10.1097/01.ico.0000244875.92879.1a . 17172891 10.1097/01.ico.0000244875.92879.1a

[14] Le Q, Zhou X, Ge L, Wu L, Hong J, Xu J. Impact of dry eye syndrome on vision-related quality of life in a non-clinic-based general population. BMC ophthalmology. 2012;12:22 Epub 2012/07/18. doi: 10.1186/1471-2415-12-22 ; PubMed Central PMCID: PMCPmc3437197. 22799274 10.1186/1471-2415-12-22 PMC3437197

[15] Mertzanis P, Abetz L, Rajagopalan K, Espindle D, Chalmers R, Snyder C, et al The relative burden of dry eye in patients' lives: comparisons to a U.S. normative sample. Investigative ophthalmology & visual science. 2005;46(1):46–50. Epub 2004/12/30. doi: 10.1167/iovs.03-0915 . 15623753 10.1167/iovs.03-0915

[16] Miljanovic B, Dana R, Sullivan DA, Schaumberg DA. Impact of dry eye syndrome on vision-related quality of life. American journal of ophthalmology. 2007;143(3):409–15. Epub 2007/02/24. doi: 10.1016/j.ajo.2006.11.060 ; PubMed Central PMCID: PMCPmc1847608. 17317388 10.1016/j.ajo.2006.11.060 PMC1847608

[17] Nichols KK, Mitchell GL, Zadnik K. Performance and repeatability of the NEI-VFQ-25 in patients with dry eye. Cornea. 2002;21(6):578–83. Epub 2002/07/20. . 12131034 10.1097/00003226-200208000-00009

[18] Schiffman RM, Walt JG, Jacobsen G, Doyle JJ, Lebovics G, Sumner W. Utility assessment among patients with dry eye disease. Ophthalmology. 2003;110(7):1412–9. Epub 2003/07/18. doi: 10.1016/S0161-6420(03)00462-7 . 12867401 10.1016/S0161-6420(03)00462-7

[19] Li M, Gong L, Sun X, Chapin WJ. Anxiety and depression in patients with dry eye syndrome. Current eye research. 2011;36(1):1–7. Epub 2010/12/23. doi: 10.3109/02713683.2010.519850 . 21174591 10.3109/02713683.2010.519850

[20] Tounaka K, Yuki K, Kouyama K, Abe T, Tsubota K, Kawabe H, et al Dry eye disease is associated with deterioration of mental health in male Japanese university staff. The Tohoku journal of experimental medicine. 2014;233(3):215–20. Epub 2014/07/25. . 25055758 10.1620/tjem.233.215

[21] Vehof J, Kozareva D, Hysi PG, Hammond CJ. Prevalence and risk factors of dry eye disease in a British female cohort. The British journal of ophthalmology. 2014;98(12):1712–7. Epub 2014/09/05. doi: 10.1136/bjophthalmol-2014-305201 . 25185440 10.1136/bjophthalmol-2014-305201

[22] Ratcliffe GE, Enns MW, Belik SL, Sareen J. Chronic pain conditions and suicidal ideation and suicide attempts: an epidemiologic perspective. The Clinical journal of pain. 2008;24(3):204–10. Epub 2008/02/22. doi: 10.1097/AJP.0b013e31815ca2a3 . 18287825 10.1097/AJP.0b013e31815ca2a3

[23] Kweon S, Kim Y, Jang MJ, Kim Y, Kim K, Choi S, et al Data resource profile: the Korea National Health and Nutrition Examination Survey (KNHANES). International journal of epidemiology. 2014;43(1):69–77. Epub 2014/03/04. doi: 10.1093/ije/dyt228 ; PubMed Central PMCID: PMCPmc3937975. 24585853 10.1093/ije/dyt228 PMC3937975

[24] Kim KW, Han SB, Han ER, Woo SJ, Lee JJ, Yoon JC, et al Association between depression and dry eye disease in an elderly population. Investigative ophthalmology & visual science. 2011;52(11):7954–8. Epub 2011/09/29. doi: 10.1167/iovs.11-8050 . 21896858 10.1167/iovs.11-8050

[25] Moss SE, Klein R, Klein BE. Long-term incidence of dry eye in an older population. Optometry and vision science: official publication of the American Academy of Optometry. 2008;85(8):668–74. Epub 2008/08/05. doi: 10.1097/OPX.0b013e318181a947 . 18677233 10.1097/OPX.0b013e318181a947

[26] Moss SE, Klein R, Klein BE. Prevalence of and risk factors for dry eye syndrome. Archives of ophthalmology. 2000;118(9):1264–8. Epub 2000/09/12. . 10980773 10.1001/archopht.118.9.1264

[27] Oden NL, Lilienfeld DE, Lemp MA, Nelson JD, Ederer F. Sensitivity and specificity of a screening questionnaire for dry eye. Advances in experimental medicine and biology. 1998;438:807–20. Epub 1998/06/23. . 9634971 10.1007/978-1-4615-5359-5_113

[28] Labbe A, Wang YX, Jie Y, Baudouin C, Jonas JB, Xu L. Dry eye disease, dry eye symptoms and depression: the Beijing Eye Study. The British journal of ophthalmology. 2013;97(11):1399–403. Epub 2013/09/10. doi: 10.1136/bjophthalmol-2013-303838 . 24013959 10.1136/bjophthalmol-2013-303838

[29] Oquendo MA, Mann JJ. Suicidal behavior: a developmental perspective. The Psychiatric clinics of North America. 2008;31(2):xiii–xvi. Epub 2008/04/29. doi: 10.1016/j.psc.2008.03.001 ; PubMed Central PMCID: PMCPmc4302391. 18439441 10.1016/j.psc.2008.03.001 PMC4302391

[30] Nock MK, Green JG, Hwang I, McLaughlin KA, Sampson NA, Zaslavsky AM, et al Prevalence, correlates, and treatment of lifetime suicidal behavior among adolescents: results from the National Comorbidity Survey Replication Adolescent Supplement. JAMA psychiatry. 2013;70(3):300–10. Epub 2013/01/11. doi: 10.1001/2013.jamapsychiatry.55 ; PubMed Central PMCID: PMCPmc3886236. 23303463 10.1001/2013.jamapsychiatry.55 PMC3886236

[31] Fine PG. Long-term consequences of chronic pain: mounting evidence for pain as a neurological disease and parallels with other chronic disease states. Pain medicine (Malden, Mass). 2011;12(7):996–1004. Epub 2011/07/15. doi: 10.1111/j.1526-4637.2011.01187.x . 21752179 10.1111/j.1526-4637.2011.01187.x

[32] Ohayon MM, Schatzberg AF. Using chronic pain to predict depressive morbidity in the general population. Archives of general psychiatry. 2003;60(1):39–47. Epub 2003/01/07. . 12511171 10.1001/archpsyc.60.1.39

[33] Pouyeh B, Viteri E, Feuer W, Lee DJ, Florez H, Fabian JA, et al Impact of ocular surface symptoms on quality of life in a United States veterans affairs population. American journal of ophthalmology. 2012;153(6):1061–66.e3. Epub 2012/02/15. doi: 10.1016/j.ajo.2011.11.030 . 22330309 10.1016/j.ajo.2011.11.030

[34] Racine M, Choiniere M, Nielson WR. Predictors of suicidal ideation in chronic pain patients: an exploratory study. The Clinical journal of pain. 2014;30(5):371–8. Epub 2013/07/28. doi: 10.1097/AJP.0b013e31829e9d4d . 23887336 10.1097/AJP.0b013e31829e9d4d

[35] Tang NK, Crane C. Suicidality in chronic pain: a review of the prevalence, risk factors and psychological links. Psychological medicine. 2006;36(5):575–86. Epub 2006/01/20. doi: 10.1017/S0033291705006859 . 16420727 10.1017/S0033291705006859

[36] Kim JH, Kwon JW. The impact of health-related quality of life on suicidal ideation and suicide attempts among Korean older adults. Journal of gerontological nursing. 2012;38(11):48–59. Epub 2012/10/17. doi: 10.3928/00989134-20121003-01 . 23066679 10.3928/00989134-20121003-01

[37] Pompili M, Lester D, Innamorati M, De Pisa E, Amore M, Ferrara C, et al Quality of life and suicide risk in patients with diabetes mellitus. Psychosomatics. 2009;50(1):16–23. Epub 2009/02/14. doi: 10.1176/appi.psy.50.1.16 . 19213968 10.1176/appi.psy.50.1.16

[38] Hyon JY, Kim HM, Lee D, Chung ES, Song JS, Choi CY, et al Korean guidelines for the diagnosis and management of dry eye: development and validation of clinical efficacy. Korean journal of ophthalmology: KJO. 2014;28(3):197–206. Epub 2014/06/03. doi: 10.3341/kjo.2014.28.3.197 ; PubMed Central PMCID: PMCPmc4038724. 24882952 10.3341/kjo.2014.28.3.197 PMC4038724

[39] Foulks GN, Forstot SL, Donshik PC, Forstot JZ, Goldstein MH, Lemp MA, et al Clinical guidelines for management of dry eye associated with Sjogren disease. The ocular surface. 2015;13(2):118–32. Epub 2015/04/18. doi: 10.1016/j.jtos.2014.12.001 . 25881996 10.1016/j.jtos.2014.12.001

[40] Chung JH, Moon K, Kim do H, Min JW, Kim TH, Hwang HJ. Suicidal ideation and suicide attempts among diabetes mellitus: the Korea National Health and Nutrition Examination Survey (KNHANES IV, V) from 2007 to 2012. Journal of psychosomatic research. 2014;77(6):457–61. Epub 2014/09/27. doi: 10.1016/j.jpsychores.2014.08.008 . 25258359 10.1016/j.jpsychores.2014.08.008

[41] Yoon CG, Bae KJ, Kang MY, Yoon JH. Is suicidal ideation linked to working hours and shift work in Korea? Journal of occupational health. 2015 Epub 2015/03/11. doi: 10.1539/joh.14-0237-OA . 25752659 10.1539/joh.14-0237-OA

[42] Lucas M, O'Reilly EJ, Mirzaei F, Okereke OI, Unger L, Miller M, et al Cigarette smoking and completed suicide: results from 3 prospective cohorts of American adults. Journal of affective disorders. 2013;151(3):1053–8. Epub 2013/09/24. doi: 10.1016/j.jad.2013.08.033 ; PubMed Central PMCID: PMCPmc3881308. 24055118 10.1016/j.jad.2013.08.033 PMC3881308

[43] Kim Y, Myung W, Won HH, Shim S, Jeon HJ, Choi J, et al Association between Air Pollution and Suicide in South Korea: A Nationwide Study. PloS one. 2015;10(2):e0117929 Epub 2015/02/19. doi: 10.1371/journal.pone.0117929 ; PubMed Central PMCID: PMCPmc4333123. 25693115 10.1371/journal.pone.0117929 PMC4333123

[44] Bernert RA, Joiner TE. Sleep disturbances and suicide risk: a review of the literature. Neuropsychiatric disease and treatment. 2007;2007:735. 10.2147/ndt.s1248 PMC2656315 19300608

[45] McCall WV. Insomnia is a risk factor for suicide—What are the next steps? Sleep. 2011;34(9):1149 doi: 10.5665/SLEEP.1222 21886349 10.5665/SLEEP.1222 PMC3157653

[46] Pigeon WR, Pinquart M, Conner K. Meta-analysis of sleep disturbance and suicidal thoughts and behaviors. 2012 doi: 10.4088/JCP.11r07586 23059158 10.4088/JCP.11r07586

[47] Ayaki M, Kawashima M, Negishi K, Tsubota K. High prevalence of sleep and mood disorders in dry eye patients: survey of 1,000 eye clinic visitors. Neuropsychiatric disease and treatment. 2015;11:889 doi: 10.2147/NDT.S81515 25848288 10.2147/NDT.S81515 PMC4386766

[48] Galor A, Feuer W, Lee DJ, Florez H, Carter D, Pouyeh B, et al Prevalence and risk factors of dry eye syndrome in a United States veterans affairs population. American journal of ophthalmology. 2011;152(3):377–84. e2. doi: 10.1016/j.ajo.2011.02.026 21684522 10.1016/j.ajo.2011.02.026 PMC4113967

[49] Wen W, Wu Y, Chen Y, Gong L, Li M, Chen X, et al Dry eye disease in patients with depressive and anxiety disorders in Shanghai. Cornea. 2012;31(6):686–92. Epub 2012/03/03. doi: 10.1097/ICO.0b013e3182261590 . 22382595 10.1097/ICO.0b013e3182261590

[50] Gulati A, Sullivan R, Buring JE, Sullivan DA, Dana R, Schaumberg DA. Validation and repeatability of a short questionnaire for dry eye syndrome. American journal of ophthalmology. 2006;142(1):125–31. Epub 2006/07/04. doi: 10.1016/j.ajo.2006.02.038 . 16815260 10.1016/j.ajo.2006.02.038

