# Supplementary material for: Association between dry eye symptoms and suicidal ideation in a Korean adult population
Source: PLoS One. 2018 Jun 20;13(6):e0199131. doi: 10.1371/journal.pone.0199131 (PMC6010274; doi:10.1371/journal.pone.0199131)
Supplement: S1 Table — (DOCX) [file pone.0199131.s001.docx]

**Supporting information**

S1 Table. The results of multivariable logistic regression analyses for depression and suicidal ideation with DED diagnosis.

| Variables | Depression diagnosis | | Suicidal ideation | |
| --- | --- | --- | --- | --- |
|  | Unadjusted  OR (95% CI) | Adjusted ^a^  OR (95% CI) | Unadjusted  OR (95% CI) | Adjusted ^a^  OR (95% CI) |
| Age | 1.01 (1.01-1.02) | 1.01 (1.01-1.02) | 1.02 (1.01-1.02) | 1.01 (1.01-1.02) |
| Body mass index | 0.99 (0.97-1.01) | 0.99 (0.97-1.02) | 1.01 (0.99-1.03) | 1.01 (0.99-1.03) |
| Sex |  |  |  |  |
| Men | 1.00 | 1.00 | 1.00 | 1.00 |
| Women | 3.29 (2.90-3.75) | 5.66 (4.56-7.03) | 2.06 (1.84-2.31) | 3.62 (3.05-4.29) |
| Alcohol consumption |  |  |  |  |
| None | 1.00 | 1.00 | 1.00 | 1.00 |
| ≤ 1 time/week | 0.65 (0.57-0.75) | 0.89 (0.75-1.05) | 0.75 (0.65-0.85) | 0.97 (0.84-1.12) |
| ≥ 2 times/week | 0.57 (0.49-0.67) | 1.14 (0.91-1.43) | 0.80 (0.68-0.93) | 1.17 (0.96-1.41) |
| Smoking behavior |  |  |  |  |
| None | 1.00 | 1.00 | 1.00 | 1.00 |
| Former | 0.69 (0.59-0.80) | 1.71 (1.36-2.15) | 0.83 (0.72-0.96) | 1.78 (1.48-2.13) |
| Current | 0.58 (0.50-0.67) | 1.80 (1.43-2.27) | 1.00 (0.87-1.15) | 2.50 (2.08-3.02) |
| Physical activity |  |  |  |  |
| No | 1.00 | 1.00 | 1.00 | 1.00 |
| ≥ 1 days/week | 0.92 (0.82-1.05) | 1.11 (0.95-1.29) | 0.84 (0.75-0.95) | 0.97 (0.86-1.09) |
| Major CVD ^b^ |  |  |  |  |
| No | 1.00 | 1.00 | 1.00 | 1.00 |
| Yes | 2.29 (1.76-2.99) | 2.61 (1.64-4.16) | 2.08 (1.56-2.76) | 1.82 (1.35-2.47) |
| Cancer |  |  |  |  |
| No | 1.00 | 1.00 | 1.00 | 1.00 |
| Yes | 1.94 (1.51-2.50) | 1.75 (1.26-2.44) | 1.46 (1.14-1.88) | 1.23 (0.95-1.60) |
| DED diagnosis |  |  |  |  |
| No | 1.00 | 1.00 | 1.00 | 1.00 |
| Yes | 1.68 (1.43-1.98) | 1.31 (1.11-1.56) | 1.38 (1.16-1.64) | 1.21 (1.02-1.43) |

DED: dry eye disease, CVD: cardiovascular diseases

^a^ Adjusted model includes all variables in the table.

^b^ Major CVD includes acute myocardial infarction and stroke.
