# Supplementary material for: Association between dry eye symptoms and suicidal ideation in a Korean adult population
Source: PLoS One. 2018 Jun 20;13(6):e0199131. doi: 10.1371/journal.pone.0199131 (PMC6010274; doi:10.1371/journal.pone.0199131)
Supplement: S2 Table — (DOCX) [file pone.0199131.s002.docx]

**Supporting information**

S2 Table. The results of multivariable logistic regression analyses for depression and suicidal ideation with dry eye symptoms.

| Variables | Depression diagnosis | | Suicidal ideation | |
| --- | --- | --- | --- | --- |
|  | Unadjusted  OR (95% CI) | Adjusted ^a^  OR (95% CI) | Unadjusted  OR (95% CI) | Adjusted ^a^  OR (95% CI) |
| Age | 1.01 (1.00-1.01) | 1.00 (0.99-1.01) | 1.01 (1.00-1.01) | 1.00 (0.99-1.01) |
| Body mass index | 1.00 (0.97-1.04) | 1.02 (0.98-1.06) | 1.01 (0.97-1.05) | 1.02 (0.98-1.06) |
| Sex |  |  |  |  |
| Men | 1.00 | 1.00 | 1.00 | 1.00 |
| Women | 3.27 (2.46-4.35) | 5.42 (3.69-7.96) | 3.44 (2.57-4.61) | 5.86 (3.93-8.75) |
| Alcohol consumption |  |  |  |  |
| None | 1.00 | 1.00 | 1.00 | 1.00 |
| ≤ 1 time/week | 0.63 (0.49-0.81) | 0.78 (0.59-1.02) | 0.64 (0.50-0.83) | 0.80 (0.60-1.05) |
| ≥ 2 times/week | 0.73 (0.54-0.99) | 1.31 (0.90-1.89) | 0.76 (0.56-1.03) | 1.40 (0.97-2.04) |
| Smoking behavior |  |  |  |  |
| None | 1.00 | 1.00 | 1.00 | 1.00 |
| Former | 0.76 (0.57-1.03) | 1.92 (1.35-2.75) | 0.75 (0.55-1.01) | 1.93 (1.33-2.80) |
| Current | 0.65 (0.47-0.90) | 1.80 (1.20-2.70) | 0.65 (0.47-0.91) | 1.86 (1.24-2.79) |
| Physical activity |  |  |  |  |
| No | 1.00 | 1.00 | 1.00 | 1.00 |
| ≥ 1 days/week | 0.98 (0.76-1.24) | 1.15 (0.90-1.48) | 0.96 (0.75-1.23) | 1.14 (0.88-1.46) |
| Major CVD ^b^ |  |  |  |  |
| No | 1.00 | 1.00 | 1.00 | 1.00 |
| Yes | 2.17 (1.31-3.59) | 2.32 (1.36-3.95) | 2.05 (1.20-3.51) | 2.16 (1.23-3.80) |
| Cancer |  |  |  |  |
| No | 1.00 | 1.00 | 1.00 | 1.00 |
| Yes | 2.60 (1.64-4.13) | 2.31 (1.42-3.75) | 2.61 (1.63-4.19) | 2.30 (1.40-3.80) |
| Dry eye symptoms |  |  |  |  |
| No | 1.00 | 1.00 | 1.00 | 1.00 |
| Yes | 1.77 (1.54-2.03) | 1.47 (1.28-1.70) | 1.60 (1.38-1.85) | 1.44 (1.24-1.66) |

DED: dry eye disease, CVD: cardiovascular diseases

^a^ Adjusted model includes all variables in the table.

^b^ Major CVD includes acute myocardial infarction and stroke.
