# Supplementary material for: Association between dry eye symptoms and suicidal ideation in a Korean adult population
Source: PLoS One. 2018 Jun 20;13(6):e0199131. doi: 10.1371/journal.pone.0199131 (PMC6010274; doi:10.1371/journal.pone.0199131)
Supplement: S3 Table — (DOCX) [file pone.0199131.s003.docx]

**Supporting information**

S3 Table. The association between dry eye and suicidal ideation after adjusting depressive symptoms

|  | Total | Suicidal ideation (%) | Model 1 | Model 2 |
| --- | --- | --- | --- | --- |
|  |  |  | OR (95% CI) | OR (95% CI) |
| Dry eye disease diagnosis |  |  |  |  |
| No | 14757 | 2034 (13.8) | 1.00 | 1.00 |
| Yes | 1696 | 285 (16.8) | 1.21 (1.02-1.43) | 1.12 (0.93-1.35) |
| Dry eye symptoms |  |  |  |  |
| No | 13092 | 1727 (13.2) | 1.00 | 1.00 |
| Yes | 2815 | 530 (18.8) | 1.44 (1.24-1.66) | 1.39 (1.18-1.63) |

Model 1: adjusted for age, sex, body mass index, smoking behavior, alcohol consumption, physical activity, major cardiovascular diseases, and cancer.

Model 2: adjusted for age, sex, body mass index, smoking behavior, alcohol consumption, physical activity, major cardiovascular diseases, cancer, and depressive symptoms.
